# PRAGMATIST: A tool to prioritize foot-and-mouth disease virus antigens held in vaccine banks

**DOI:** 10.3389/fvets.2022.1029075

**Published:** 2022-12-15

**Authors:** Anna B. Ludi, Melissa McLaws, Bryony Armson, Jessica Clark, Antonello Di Nardo, Krupali Parekh, Mark Henstock, Petra Muellner, Ulrich J. Muellner, Fabrizio Rosso, Joaquin M. Prada, Daniel L. Horton, David J. Paton, Keith Sumption, Donald P. King

**Affiliations:** ^1^Vesicular Disease Reference Laboratory, The Pirbright Institute, Woking, United Kingdom; ^2^The European Commission for the Control of Foot and Mouth Disease (EuFMD), Food and Agriculture Organization of the United Nations, Rome, Italy; ^3^Wellcome Centre for Integrative Parasitology, Institute of Biodiversity, Animal Health and Comparative Medicine, University of Glasgow, Glasgow, United Kingdom; ^4^Faculty of Health and Medical Sciences, School of Veterinary Medicine, University of Surrey, Guildford, United Kingdom; ^5^Epi-Interactive, Miramar, Wellington, New Zealand; ^6^School of Veterinary Science, Massey University, Palmerston North, New Zealand

**Keywords:** vaccination, vaccine matching, vaccine bank, foot and mouth disease (FMD), decision support tool, vaccine selection

## Abstract

Antigen banks have been established to supply foot-and-mouth disease virus (FMDV) vaccines at short notice to respond to incursions or upsurges in cases of FMDV infection. Multiple vaccine strains are needed to protect against specific FMDV lineages that circulate within six viral serotypes that are unevenly distributed across the world. The optimal selection of distinct antigens held in a bank must carefully balance the desire to cover these risks with the costs of purchasing and maintaining vaccine antigens. PRAGMATIST is a semi-quantitative FMD vaccine strain selection tool combining three strands of evidence: (1) estimates of the risk of incursion from specific areas (source area score); (2) estimates of the relative prevalence of FMD viral lineages in each specific area (lineage distribution score); and (3) effectiveness of each vaccine against specific FMDV lineages based on laboratory vaccine matching tests (vaccine coverage score). The output is a vaccine score, which identifies vaccine strains that best address the threats, and consequently which are the highest priority for inclusion in vaccine antigen banks. In this paper, data used to populate PRAGMATIST are described, including the results from expert elicitations regarding FMD risk and viral lineage circulation, while vaccine coverage data is provided from vaccine matching tests performed at the WRLFMD between 2011 and 2021 (*n* = 2,150). These data were tailored to working examples for three hypothetical vaccine antigen bank perspectives (Europe, North America, and Australia). The results highlight the variation in the vaccine antigens required for storage in these different regions, dependent on risk. While the tool outputs are largely robust to uncertainty in the input parameters, variation in vaccine coverage score had the most noticeable impact on the estimated risk covered by each vaccine, particularly for vaccines that provide substantial risk coverage across several lineages.

## Introduction

Foot-and-mouth disease virus (FMDV) exists as seven serotypes: O, A, C, Asia 1, SAT 1, SAT 2 and SAT 3, although serotype C has not been reported since 2004 (1, 2). The world is divided into FMD-free and endemic countries and regions (3), with virus widespread in Africa and Asia and restricted to Venezuela in South America. FMDV serotypes and strains are unevenly distributed in different parts of the world with seven geographic pools of FMDV identified (4). Each virus pool has more than one serotype, within which FMDV strains evolve and circulate (5, 6), giving rise to waves of infection and potential for periodic spread of strains beyond their pools of origin (7–9). Recent examples of FMDV strains that have spread widely are O/ME-SA/Ind-2001 (10) and A/ASIA/G-VII (11).

Prophylactic vaccination is widely used to control FMD where the virus is endemic or where incursions are highly likely (3). Vaccination is also an emergency option in response to incursions in FMD-free countries or upsurges of infection in FMD-endemic countries (12). The emergence and spread of antigenic variants within FMDV serotypes can require multiple vaccine strains, as immunity, whether induced by infection or vaccination, is serotype specific and may be weak or incomplete between antigenically divergent strains (13). The expected level of protection conferred by a vaccine is often measured by vaccine matching, an *in vitro* test which compares the seroreactivity of vaccine antisera to the vaccine strains (homologous reactivity) and the field strains (heterologous reactivity). Vaccination-challenge tests in the target species can also be undertaken to provide empirical data for vaccine performance, but wide-scale use of these *in vivo* approaches is often constrained by cost, time and animal welfare considerations.

Countries that are FMD-free take stringent measures to prevent incursions of FMD and ensure preparedness in the event of an outbreak, including provision of vaccine reserves for implementation of emergency vaccination. These strategic reserves mostly take the form of concentrated FMDV antigens frozen above liquid nitrogen, with a long shelf life, that can be rapidly thawed and formulated as ready-to-use vaccines (14). Europe and North America have established multinational vaccine banks of this type and there may be at least 20 national banks worldwide. Along with rapid formulation into final vaccine product, antigen banks have several technical advantages, such as consistency in production and quality assurance (14, 15). However, the antigens maintained in the bank must be carefully and timely selected to provide protection against the most important viral threats, balancing vaccine availability from manufacturers with the costs of carrying unused antigens. Working with FMD reference laboratories and vaccine producers, bank managers assess recent epidemiological events to determine current and future threats posed by circulating viral strains. The FAO World Reference Laboratory for FMD (WRLFMD) previously provided vaccine antigen bank recommendations on a quarterly basis, in which the most common vaccine strains were classified into high, medium, and low priority [see quarterly reports until December 2017 (WRLFMD)]. However, the criteria for determining into which category an antigen was placed were not clearly defined and these recommendations were based on European vaccine producers and threats to FMD-free European countries that may not have been appropriate for countries in other regions.

In this paper, we describe and apply a novel *Prioritization of Antigen Management with International Surveillance Tool* (PRAGMATIST) to assist vaccine bank managers in selecting which FMDV strains are most important to maintain in their vaccine bank. This tool provides a transparent, evidence-based framework to evaluate available vaccine antigens, that can be adapted according to the region at risk.

## Methods

### Design of PRAGMATIST

The decision-support tool provides a structured framework to assist vaccine bank managers to prioritize vaccine strains that are candidates for inclusion in an antigen bank. The tool combines three relevant parameters from the perspective of an antigen bank manager, namely (1) the relative likelihood of an FMD incursion from different regions of the world (source areas); (2) the prevalence of circulating FMD viral strains in these source areas (lineage distribution) and (3) the expected protection afforded by different FMD vaccines against these circulating FMD strains (vaccine coverage). The level of protection is based on the antigenic relationships defined by serological vaccine matching studies (1, 16) which could be complemented by direct evidence of protection in the field where these data are available. The lineage distributions are specific to the source regions, whilst the source area scores and vaccine availability will be specific to the country or region at risk. PRAGMATIST was initially developed and is still currently available in MS-Excel (https://www.eufmd.info/pragmatist). However, to improve accessibility and strengthen science-to-policy linkage (17), the tool has been ported to an easy-to-use interactive dashboard, with the application's scope and interface design crafted with structured input from multiple stakeholder groups, including beta testing of the application. The web-platform (www.openfmd.org/dashboard/PRAGMATIST) was developed in R Shiny (18, 19) by further adding extended functionalities using JavaScript and Cascading Style Sheets (CSS).

### Source area score (SAS)

The first step in the tool is to assign source area scores (SAS). The source areas correspond to the geographic extent of each endemic virus pool (4), with an additional area encompassing specific countries in North Africa (Morocco, Algeria, Tunisia and Libya). Long-term maintenance of FMD has not been historically documented in North Africa and therefore this region does not constitute an FMD endemic pool. However, recent introductions of diverse FMDV lineages into this region (O/ME-SA Ind-2001d in 2014–2015 (10), A/AFRICA/G-IV in 2017 (20) and O/EA-3 in 2018 and 2021 (21), pose a distinct threat to FMD-free countries in Europe.

The SAS should be populated by the vaccine bank manager (the user) and will be tailored to address the particular risks of FMD introduction into the country or region covered by the antigen bank. The user allocates 100 points among the potential source areas, giving more points to the areas they consider a higher likelihood of being the source of an incursion. A source area can be allocated zero points if it is not considered important. The tool does not prescribe how the SAS should be defined, but expert elicitation can be used, engaging those knowledgeable about transboundary trade and other risk pathways into the target region.

### Lineage distribution score (LDS)

The second step in the tool indicates the lineage distribution score (LDS) which specifies the distribution of specific FMDV lineages circulating within each source area. Viral lineages considered most important for transboundary spread are included in PRAGMATIST.

These virus strains are summarized by serotype|topotype|lineage, and for ease are hereafter referred to as lineages. In some instances, lineages are combined together to simplify the use of the tool, such as: (i) O EA-2, O EA-3, O EA-4 and O WA which are grouped as O EA or O WA; (ii) A Africa G-1 and G-IV grouped as A AFRICA; (iii) Asia 1 Sindh-08 and non-specified Asia 1 lineages grouped as Asia 1; (iv) SAT 1 I(NWZ), SAT 1 II(SEZ), SAT 1 III(WZ), and SAT 1 X grouped as SAT 1; and (v) SAT 2 I, SAT 2 II, SAT 2 III, SAT 2 IV, and SAT 2 VII grouped as SAT 2.

To define the LDS, each source area is allocated 100 points which are divided between the different FMDV lineages circulating in that area. The LDS provides an estimate of how often each lineage would be detected if 100 FMDV-infected animals were randomly selected from a source area in the previous year. The default scores set in the tool are based on data generated through FMD regional surveillance activities. These values are discussed and updated at each annual meeting of the WOAH/FAO Reference Laboratory Network (www.foot-and-mouth.org) and reviewed and reported quarterly by the WRLFMD (41). However, these scores can also be modified by the user when required.

### Lineage risk score (LRS)

The lineage risk score combines the SAS and LDS, to give an overall risk score (max possible score = 10,000) for each FMDV lineage. The LRS is calculated according to the formula:


(1) $$LRS\;=\;\sum {}_{source\;area\;1}^{source\;area\;n}\left(LDS*SAS\;\right)$$

### Vaccine coverage score (VCS)

The vaccine coverage score (VCS) reflects whether a specific FMD vaccine is likely to provide protection against each of the FMDV lineages. Consequently, a VCS is given for each combination of vaccine and viral lineage included in the tool and is calculated as the proportion of field isolates from each particular lineage that antigenically match the vaccine in question.


(2) $$\begin{array}{l} VCS\;=\;\left(\frac{Number\;of\;isolates\;that\;match\;vaccine\;strain}{Number\;of\;isolates\;tested}\;\right) \end{array}$$

These data are obtained from routine vaccine matching studies that are undertaken by the WRLFMD, where a match between a vaccine and field strain is defined as a one-way relationship value (r_1_ value) of greater than or equal to 0.3, determined by a virus neutralization test using monovalent vaccine-specific antisera (1). The VCS can be adjusted by the user if other information exists about the likelihood that a vaccine provides protection based on efficacy or effectiveness data from *in-vivo* cross-protection or field studies, respectively. For example, cross-protection vaccine-challenge studies may show that a high potency formulation of a vaccine strain may elicit satisfactory protection to a field strain despite a poor match *in-vitro* (13). Details of known studies where results may influence vaccine coverage scores are shown in the Supplementary Data (Supplementary Data Table 1).

### Vaccine score (VS)

Finally, the vaccine score (VS) is calculated according to the formula:


(3) $$VS\;=\;\sum {}_{virus\;strain\;1}^{virus\;strain\;n}\left(VCS*LRS\;\right)$$

The VS is a final score for each vaccine/lineage combination, and combines the risk posed by specific lineages to a particular region (lineage risk score) with the expected protection conferred by the vaccine (vaccine coverage score). Vaccines with the highest scores will therefore be those that provide protection against the most important FMDV threats in the region targeted by the antigen bank.

### Application of PRAGMATIST

As working examples, PRAGMATIST was populated with parameters appropriate for vaccine bank managers from three regions: Europe, North America and Australia where the SAS were obtained using a modified Delphi expert elicitation process (22). A questionnaire was administered to experts who were asked to divide 100 points between the potential source areas, with the most points going to the area(s) that posed the highest risk to the countries serviced by each region's vaccine bank. Results from the first round were summarized and discussed, and then the questionnaire was administered again in a final round. Responses were averaged to obtain the final SAS. For the European vaccine bank perspective, experts were country representatives (one per country) attending the European National Reference Laboratories for FMD Workshop in 2017. For the North America and Australia vaccine bank perspectives, experts were participants at a workshop held at the 2018 EuFMD Open Session (23).

The LDS were assigned by regional experts at the 2020 annual meeting of the WOAH/FAO Reference Laboratory Network. Finally, the VCS were populated through analysis of routine vaccine matching test data performed by the WRLFMD between 2011 and 2021, for vaccines produced by commercial vaccine companies and where reagents (vaccine strains, vaccine antisera and field strains) are available at WRLFMD for this testing.

### Sensitivity analysis

An optimisation algorithm was used to identify which sources of uncertainty in the tool's input values have the greatest impact on the prioritization of FMD vaccine antigens. There are several underlying assumptions: (i) when a vaccine is selected it reduces the risk of all matched lineages, (ii) the coverage provided by each vaccine is not additive, such that the risk posed by a lineage is only reduced by the amount equal to the highest coverage of the selected vaccines, and (iii) there is no cross-serotype reactivity.

Uncertainty was considered in all three user inputs (SAS, LDS and VCS). For SASs and LDSs, six levels of user-identified confidence were introduced: (i) “none”—chosen when the user has no confidence in the input values, (ii) “low”, (iii) “mid-low”, (iv) “mid”, (v) “mid-high”, and (vi) “high”. These categories correlate to the weighting on the variance around the input score, with the input drawn from a truncated normal distribution bound between 0 and 100, where the mean is the user stated input, a standard deviation of 1.5 and the weighting of 7.5, 6, 4.5, 3, and 1.5 or no weighting correlating to user confidence, respectively. All scores were scaled between 0 and 100 as per the tool in the non-stochastic form.

VCS uncertainty is influenced by two main factors. First, from the range of r_1_ values obtained in the vaccine matching tests when the same vaccine is matched to different examples of a given field strain (where the uncertainty is influenced by inherent variability of the vaccine matching test and antigenic diversity within each viral lineage), and second, from the number of paired tests performed for each vaccine-field strain combination. Stochasticity was therefore introduced in two steps. Step 1: for each vaccine/lineage combination, a beta distribution was fitted to capture the breadth of r_1_ values. From each distribution, *N* r_1_ values were sampled, where *N* defines the number of vaccine/lineage matching tests in the data. From these simulated values the VCS was calculated as above (equation 2). For Step 2, this VCS was then penalized depending on the number of tests that informed this score. Another draw was made from a beta distribution, this time parameterized based on mean and precision in the form.


(4) $$\begin{array}{l} Simulated\;vaccine\;coverage\;score\;=\;\beta \left(\left(\alpha *\tau \right),\left(\tau *1-\;\alpha \right)\right) \end{array}$$

Where α defines the mean value (i.e., the vaccine coverage score drawn in step 1) and τ the weighting reflecting the number of tests performed. There were seven weightings used: τ = 2 if only one test had been conducted, such that the vaccine coverage score was drawn from a uniform distribution {0,1}. τ = 4 when the number of tests were ≥1 and ≤ 10. τ = 8 when the number of tests were ≥11 and ≤ 20. τ = 16 when the number of tests were ≥21 but ≤ 40. τ = 24 when the number of tests were ≥41 and ≤ 60. τ = 32 when the number of tests were ≥61 but ≤ 80. Finally, τ = 40, when the number of tests were >80.

### Data analysis

Data analysis was performed using R (version 4.1.2) (19).

## Results

### Source area score

The SASs obtained from the expert elicitation are shown in Table 1. From the European vaccine bank perspective, the experts considered that Pool 3 posed the highest risk as the source of an incursion of FMDV, followed by North Africa, comprising 43 and 23% of the risk, respectively. For North America, Pool 1 was allocated the highest score (30%), with marginally lower values allocated to Pool 2 (24%) and Pool 3 (20%). For Australia, Pool 1 was ascribed a SAS of 70% which was much higher than any of the other potential source areas.

**Table 1:** Source area scores obtained through expert elicitation for each region.

**Source area**	**Europe**	**North** **America**	**Australia**
Pool 1 [Southeast/ Central/ East Asia]	11	30	70
Pool 2 [South Asia]	8	24	10
Pool 3 [West Eurasia and Middle East]	43	20	5
North Africa	23	10	5
Pool 4 [Eastern Africa]	4	4	2
Pool 5 [West/ Central Africa]	5	4	2
Pool 6 [Southern Africa]	3	4	3
Pool 7 [South America]	3	4	3
Total	100	100	100

### Lineage distribution score and lineage risk score

**Figure 1:**
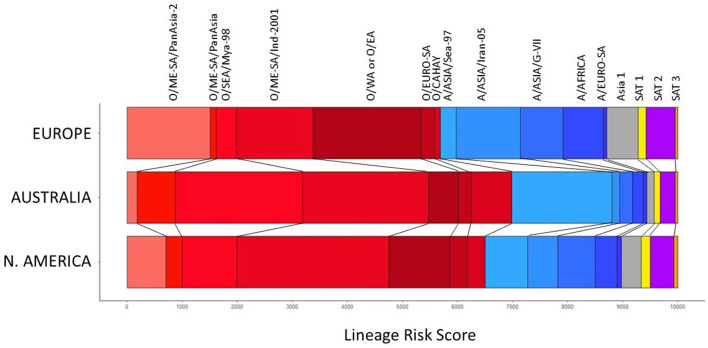
Total lineage risk scores per serotype/topotype/lineage for each vaccine bank perspective.

The LDSs determined by experts that attended the 2020 WOAH/FAO Reference Laboratory Network meeting, are shown in Table 2. The resulting lineage risk scores are given for each vaccine bank perspective in Figure 1. For Europe, O EA or O WA had the highest LRS, for North America, O ME-SA Ind-2001 had the highest LRS, and for Australia, O SEA Mya-98 and O ME-SA Ind-2001 had the highest LRS.

**Table 2:** Lineage distribution scores for each source area.

**Serotype** **|Topotype| Lineage**	**Pool 1 [Southeast/ Central/ East Asia]**	**Pool 2 [South Asia]**	**Pool 3 [West Eurasia and Middle East]**	**North Africa**	**Pool 4 [Eastern Africa]**	**Pool 5 [West/ Central Africa]**	**Pool 6 [Southern Africa]**	**Pool 7 [South America]**
O ME-SA PanAsia-2	-	-	35	-	-	-	-	-
O ME-SA PanAsia	10	-	-	-	-	-	-	-
O SEA Mya-98	33	-	-	-	-	-	-	-
O ME-SA Ind2001	20	80	7	10	-	-	-	-
O EA or O WA	-	-	3	55	55	70	-	-
O EURO-SA	-	-	-	-	-	-	-	80
O CATHAY	10.5	-	-	-	-	-	-	-
A ASIA Sea-97	26	-	-	-	-	-	-	-
A ASIA Iran-05	-	-	27	-	-	-	-	-
A ASIA G-VII	-	16	15	-	-	-	-	-
A AFRICA	-	-	-	25	22	15	-	-
A EURO-SA	-	-	-	-	-	-	-	20
Asia 1	0.5	4	12.5	-	-	-	-	-
SAT 1	-	-	-	-	8	5	27	-
SAT 2	-	-	0.5	10	14	10	57	-
SAT 3	-	-	-	-	1	-	16	-
Total	100	100	100	100	100	100	100	100

### Vaccine coverage score

**Figure 2:**
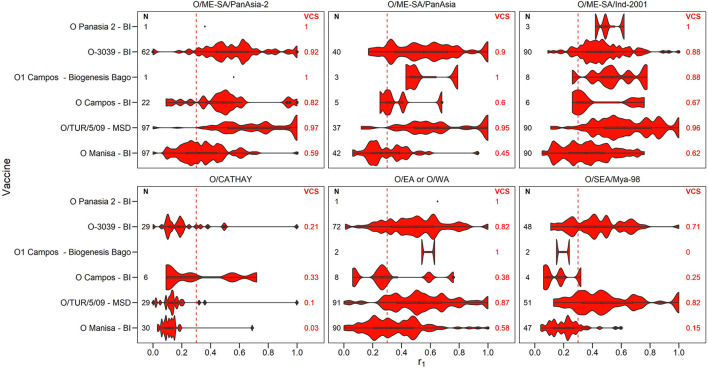
Violin plots showing results of vaccine matching tests performed at the WRLFMD between 2011 and 2021, for each vaccine/lineage combination for serotype O. Resulting vaccine coverage scores (VCS) are labeled on the right in red, and the number of tests performed are labeled on the left in black. The red dashed line shows the r_1_ cut-off of 0.3 indicative of an effective vaccine match. Values for lineage EURO-SA are not shown as there was only one test performed for each of the vaccines O Campos (BI), O1 Manisa (BI MSD) and O/TUR/5/09 (MSD), with all r_1_ values being above 0.3.

**Figure 3:**
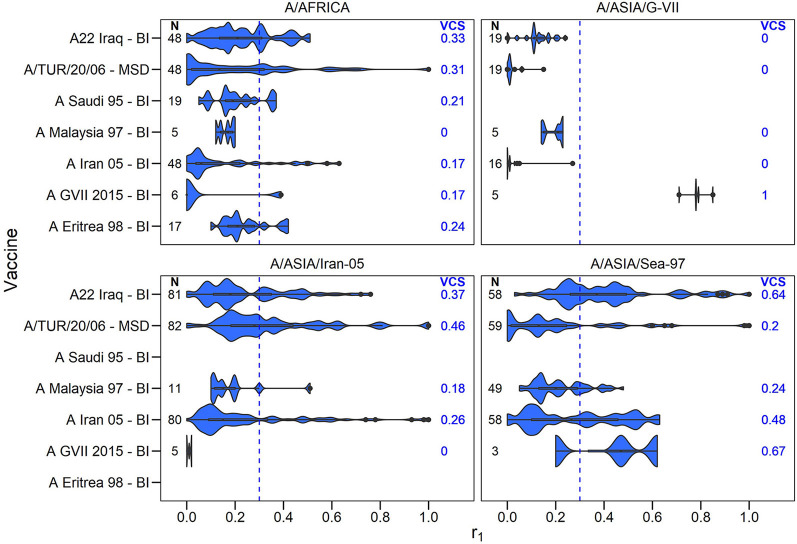
Violin plots showing results of vaccine matching tests performed at the WRLFMD between 2011 and 2021, for each vaccine/lineage combination for serotype A. Resulting vaccine coverage scores are labeled on the right in blue, and the number of tests performed are labeled on the left in black. Blue dashed line shows the cut off of 0.3 r_1_ indicative of an effective vaccine match.

**Figure 4:**
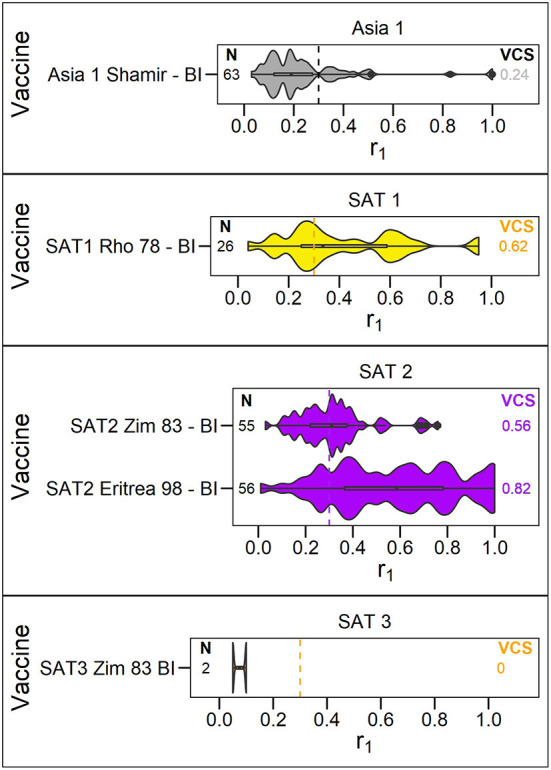
Violin plots showing results of vaccine matching tests performed at the WRLFMD between 2011 and 2021, for each vaccine/lineage combination for serotypes Asia 1, and SAT 1–3. Resulting vaccine coverage scores are labeled on the right, and the number of tests performed are labeled on the left in black. Gray dashed line shows the cut off of 0.3 r_1_ indicative of an effective vaccine match.

A summary of the r_1_ values resulting from vaccine matching tests performed at the WRLFMD between 2011 and 2021 is shown in the violin plots in Figures 2–4, along with the number of tests performed and resulting VCSs. These VCSs are displayed in the editable summary in the PRAGMATIST. Further details regarding the number of samples collected per year and region are provided in Supplementary Table 2 and Supplementary Figures 1a-d. Only one sample from South America was obtained for vaccine matching.

The number of vaccine matching tests performed per vaccine/lineage combination ranged from a minimum of 1 and maximum of 97 for serotype O, 1 – 82 for serotype A, 63 tests for Asia 1, 26 for SAT 1, 55-56 for SAT 2, and with only 2 tests for SAT 3. Not all vaccines were tested against all lineages (Figures 2–4). These figures display the range of r_1_ values that have been observed for each vaccine/lineage combination.

A VCS of 1.0 was reported for 9 vaccine/lineage combinations suggesting a good antigenic match, however, confidence in these results is poor due to the small sample size (≤ 5). For serotype O, the O/ME-SA/PanAsia-2, O/ME-SA/PanAsia, O/ME-SA/Ind-2001, O EA or WA, and O EURO-SA lineages were generally well matched against the vaccines tested (Figure 2). For the O EURO-SA lineage all r_1_ values were above 0.3 (VCS = 1.0), however only one vaccine matching test was performed for this lineage against each of the vaccines: O Campos (BI), O1 Manisa (BI MSD) and O/TUR/5/09 (MSD), and therefore confidence in the VCS is low. Additionally, only a small number of vaccine matching tests were performed for the O-Panasia 2 (BI) vaccine strain. The O CATHAY lineage was the least well matched with any of the vaccines tested.

The performance of the serotype A vaccines against the different lineages was generally poor, however vaccine coverage scores were generally higher against the A/ASIA/SEA-97 lineage (Figure 3). Only the A G-VII (BI) vaccine demonstrated matching against the A/ASIA/G-VII lineage from South Asia, with all r_1_ values > = 0.3 (VCS = 1.00, sample size = 5). No samples from the A/EURO-SA lineage were obtained for vaccine matching.

The Asia1 Shamir (BI MSD) vaccine and SAT3 ZIM 83 (BI) vaccine were poorly matched to Asia 1 (VCS = 0.24) and SAT 3 (VCS = 0.0) field strains, respectively, with only two vaccine matching tests performed for SAT 3. For SAT 1 and SAT 2, variability was observed for each of the vaccines reflecting the variability in field strains, but with over 50% of isolates tested matching (VCS for SAT1 Rho 78 = 0.62, SAT2 ZIM 83 = 0.56, SAT2 Eritrea 98 = 0.82, Figure 4).

### Vaccine scores

**Figure 5:**
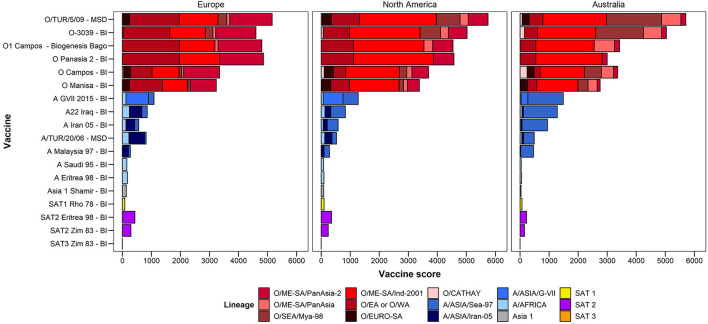
Vaccine scores for each vaccine–lineage combination.

Figure 5 summarizes the vaccine scores for each vaccine/lineage combination, for each of the three vaccine bank perspectives. The vaccine scores can be utilized to assist in vaccine selection for each vaccine bank. For example, for serotype O, the O/TUR/5/09 (MSD) vaccine had the highest vaccine score for all three vaccine bank perspectives (Europe, North America, and Australia), although the lineage-specific components differ according to the LRSs for each of the three antigen banks. Similar data highlighting the highest priority vaccine antigens and their coverage against the risks posed by different viral lineages are also presented in the figure for other FMD serotypes.

### Uncertainty

**Figure 6:**
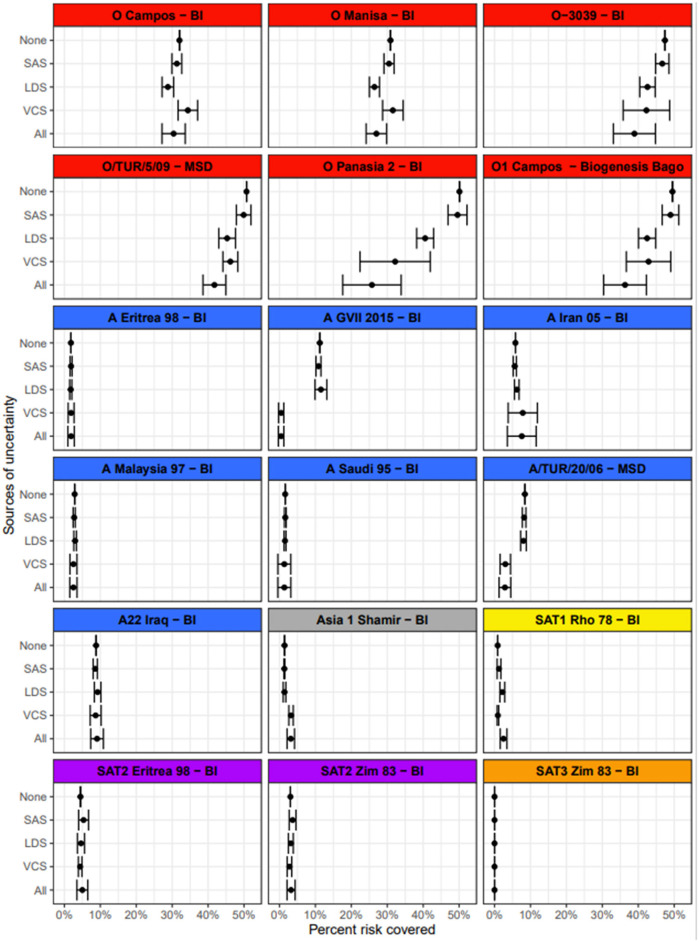
The mean and standard deviation for the percentage risk coverage out of the total risk needing to be covered, for each vaccine. The sources of variance are broken down; none indicates how the tool would work as it is, in the absence of any stochasticity. SAS is uncertainty in the source area scores (set to “mid” here), with no variation introduced from other inputs. LDS is uncertainty in the lineage distribution scores (set to “mid” here), with no variation introduced from other inputs. VCS is variation in the vaccine coverage scores based on the breadth of r_1_ values from vaccine matching tests and the number of tests performed, with no variation introduced from other inputs. All indicates uncertainty in all parameters, using a “mid” level of uncertainty in the LDS and SAS.

For the purpose of illustration, the impact of uncertainty in the input values was demonstrated using the European values for the SAS and “mid” levels of uncertainty in the input parameters. Results indicate that identifying which vaccines cover the most risk is largely robust to uncertainty in the input values (Figure 6). Uncertainty in the vaccine coverage score had the greatest impact on the percentage of the total risk covered. This was particularly obvious for vaccines such as O-3039 (BI) that cover a large proportion of the risk. In the simulation of the vaccine coverage score, each vaccine/lineage combination had a wide range of empirical r_1_ values underlying the distribution from which the score was drawn from, and then the simulated score was penalized depending on the number of tests. Consequently, uncertainty was compounded for vaccines that protect against multiple lineages. This was also true when considering uncertainty in all three input parameters at the same time [Figure 6 (all)], where the inclusion of uncertainty reduced the estimated percentage risk covered, notably for vaccines that covered a substantial portion of risk. When vaccines do not cover substantial proportions of the risk, the variation in input data for the VCS has little effect. All levels of uncertainty for all regions are shown in Supplementary Data Files 2a–b, 3a–c. In summary, vaccine choice was more tolerant to uncertainty in the SAS and LDS, rather than the VCS.

## Discussion

PRAGMATIST provides a transparent and accessible, evidence-based decision support tool to assist FMD vaccine bank managers to determine which vaccine antigens are highest priority for storage. This is achieved through combining the scores for three key criteria: the level of threat posed by different endemic regions (SAS), the prevalence of different FMD viral lineages in those regions (LDS), and the effectiveness of vaccines against those viral lineages, based on *in vitro* vaccine matching testing (VCS). Combining these scores enables vaccine bank managers to select those vaccines that should be most effective against the current threats for that region, based on the available evidence (Table 3).

**Table 3:** Using PRAGMATIST.

**PRAGMATIST parameter**	**How to complete each parameter of PRAGMATIST**	**Considerations/limitations**	**Potential modifications that can be made by the user**
Source area score (SAS)	The user allocates 100 points among the different FMD endemic source areas according to the risk of FMD introduction into the target country/region. This can be informed by expert elicitation.	Expert opinion may differ depending on their knowledge of relevant factors, such as transboundary trade, risk pathways and farm management practices. This parameter is difficult to quantify accurately, due to the number of determinants, the ever-changing situation and gaps in the information required.	Source areas could be tailored to accommodate a different spectrum of countries. Specific scores could be informed by local knowledge or qualitative/ quantitative risk-assessment tools.
Lineage distribution score (LDS)	Each source area is allocated 100 points which are divided between the different FMDV lineages circulating in that area (i.e., relative frequency of these lineages if 100 FMD infected animals were to be randomly sampled). Default scores are based on data generated through FMD regional surveillance activities, updated at each annual WOAH/FAO Reference Laboratory Network meeting (www.foot-and-mouth.org).	Up to date knowledge of circulating viral lineages in each source area is required. Continued viral evolution and emergence of new strains with novel antigenic phenotypes. A lack of disease reporting in some areas may mean that some viral lineages are under-reported. Detailed molecular epidemiological data may not be made widely available/communicated. The grouping of viral lineages as presented (e.g., grouping O EA & O WA) may not represent the diversity of FMDV lineages present in an important source area (for example, sparse surveillance of some of the African endemic pools currently constrains the level granularity that can be achieved).	Expert elicitation by other methods. Grouping of viral lineages can be separated/changed when new data becomes available.
Vaccine coverage score (VCS)	The vaccine coverage score is calculated as the proportion of field isolates from each lineage that antigenically match the vaccine in question (r_1_ value of ≥0.3). Default scores are based on routine vaccine matching studies undertaken by the WRLFMD.	The r_1_ values may lack precision due to incomplete repeatability and reproducibility of neutralization tests. Vaccine matching data does not provide a guarantee that protection will be afforded against a particular lineage, as various factors may affect vaccine efficacy. Not all vaccines are tested against all lineages, and the number of vaccine matching tests performed for some vaccine/lineage combinations may be low. Uncertainty in the vaccine coverage score has the greatest impact on the percentage of the total risk covered and is more obvious for vaccines that cover more risk. Vaccine matching results can vary for different isolates within the same lineage. The grouping of viral lineages as presented may not be appropriate (for example, grouping O EA and O WA together).	Include vaccine matching data from alternative laboratories. The score may be adjusted based on additional information: • Inclusion of vaccine matching data for additional vaccine strains (if local data are available), • Vaccine efficacy data from *in-vivo* cross-protection vaccine-challenge experiments, • Data from field vaccine evaluation studies, • Alternative indicators of protection from e.g., sequence-based approaches, • Vaccine batch-specific data, • Data derived from studies of the performance of polyvalent vaccines. Sub-divide vaccine matching data by lineage geographically or chronologically.
Vaccine score	The vaccine score combines the lineage risk score with the vaccine coverage score. Vaccines with the highest scores will be those that provide protection against the most important FMDV threats.	Choosing only vaccines with the highest scores may provide redundant protection. A low score may indicate a lack of vaccine matching data, rather than a lack of protection.	Sharing arrangements between different vaccine banks may allow for synergistic vaccine selection based on complementary choices.

PRAGMATIST is a simple-to-use tool which is provided with pre-populated values for LDS and VCS, based on expert opinion from the WOAH/FAO FMD Reference Laboratory Network and vaccine matching data from the WRLFMD, respectively. However, the user has complete control to adjust these inputs to accommodate local knowledge and up-to-date epidemiological information.

The outputs from the tool are tailored for different geographical perspectives by the user who inputs a SAS that addresses the likelihood an FMD incursion will originate from different geographical regions. These threats might vary according to the level and complexity of inter-regional connectivity (such as those epidemiological factors associated with geographic proximity, animal movements, plus legal and illegal trade of livestock and animal products, cultural and religious practices), the weight of infection in the source area (e.g. susceptible population sizes, incidence of infection) and the effectiveness of cross-border risk mitigation measures (24, 25). These parameters are difficult to quantify precisely due to their dynamic nature, the multiplicity of determinants and circumstances, the chance nature of transmission opportunities and the many gaps in required information. Therefore, for PRAGMATIST, assessment based on expert knowledge has been used to estimate the SAS, which was deemed appropriate given the expert elicitation process used, the participants involved, and that uncertainty in the SAS had a smaller effect on the outcome compared to the LDS and VCS. Several tools are available to perform more structured, qualitative or quantitative assessments of exotic animal disease incursion risk (26–29). Notably, Condoleo *et al* (30) used the progress of countries along the FMD Progressive Control Pathway (PCP-FMD) (31) to rank the FMD hazard that they pose. Additionally, The European Commission for the Control of Foot and Mouth Disease's (EuFMD) risk monitoring tool (32) combines the disease status, transmission pathways and inter-country connections to provide a rapid assessment of which countries pose the greatest incursion risk for FMD and similar transboundary animal (FAST) diseases. In the future, these tools could inform or link with PRAGMATIST to provide improved justification for SAS values (Table 3).

The LDS requires information on the relative prevalence of serotypes and viral lineages in each viral pool. Knowledge of this is incomplete, due to under-reporting and continuous viral evolution leading to the emergence of new strains. Like other highly contagious diseases, FMD incidence is often cyclical, associated with opportunities for virus spread and the waxing and waning of population immunity (8, 9). Additionally, it is likely that there may be inherent characteristics of particular viral lineages that facilitate their transmissibility. These factors are not considered in this tool, but an ability to transfer between geographical “virus pools” could be a warning sign that a strain poses a greater threat of incursion. For simplicity, PRAGMATIST currently combines the risks associated with certain FMD viral lineages together in the LDS for example those from East and West Africa. Although the African endemic pools provide a low contribution to the SASs in the worked examples in the paper, future development of the tool will inevitably consider the antigenic diversity that exists across the African FMDV serotypes and the suitability of vaccines to provide protection against these lineages.

The lineage risk score provides an overall score taking into consideration the relative prevalence of each viral lineage in each virus pool, and the risk of an incursion of that lineage. For all three vaccine bank perspectives the risk from Asia 1, SAT 1, SAT 2 and SAT 3 was less than 1/5^th^ of the total lineage risk, with the majority coming from serotype A and O lineages. Indeed, these two serotypes are the most prevalent, with the widest known geographical distribution. Individual lineages scored differently between the vaccine bank perspectives, as expected, due to the threat of circulating viral lineages in each region. For example, O/SEA/Mya-98 and A/Asia/SEA-97 scored highly from the Australian perspective, reflecting their prominence in pool 1 which is considered the most highly connected source of risk for FMD for Australia, while the score was lower from the European perspective. From the North American perspective, O/ME-SA/Ind-2001 had the largest lineage risk score, reflecting its circulation in pools 1, 2 and 3 as well as in North Africa, all of which are considered important source areas for North America.

The default VCSs included in the tool are based on routine *in vitro* vaccine matching tests performed by the WRLFMD. In calculating these VCSs, previously unpublished vaccine matching data from the WRLFMD from tests performed between 2011 and 21 has been collated for the first time, comprising 2,150 individual data points for field strain/vaccine pairs (1207 for serotype O, 741 for serotype A, 63 for serotype Asia 1, 26 for serotype SAT, 111 for serotype SAT 2 and 2 for serotype SAT 3, respectively). These vaccine matching results help to select antigenically appropriate vaccine strains, and the data presented in this report highlight where individual vaccines are consistently well-matched against field isolates. These data also reveal where the available vaccines indicate the potential for poor protection, where most of the r_1_ values are below 0.3, such as for the O/CATHAY topotype. Indeed, these data can identify where there may be gaps in antigenic vaccine coverage, for example, poor matching data for the emerging A/ASIA/G-VII lineage led to the recent development of new specific vaccine strains to cover the spread of this lineage in the Middle East (11, 33, 34). The data reveal that vaccine matching test results can vary substantially for different isolates within the same lineage. It is uncertain the extent to which this variability is attributable to the low repeatability of vaccine matching tests (35) vs. inherent antigenic differences between the isolates themselves. Analysis for temporal trends in the variability of vaccine matching results might reveal evidence for change accumulating through evolution. In the current version of PRAGMATIST, as mentioned above, certain FMD viral topotypes/lineages are grouped together, such as the O/EA/1-4 and O/WA topotypes, and SAT 2 topotypes, with the resulting VCS based on this grouping. Indeed, grouping topotypes/lineages differently, or not at all, would result in differing VCSs, however, the number of vaccine matching tests performed for each grouping would decrease, potentially reducing confidence in these scores.

PRAGMATIST users should apply caution if only a few matching tests have been performed, which was the case for several lineage-vaccine combinations in our study. Ideally, for a given serotype, all available vaccines should be tested against all circulating lineages, using many original isolates. However, availability of field isolates and vaccine strains at the WRLFMD limits the amount of possible testing combinations. For some field strains, isolates from multiple sources are available, whilst for others, only a single isolate may have been submitted for testing, despite efforts made to facilitate submission of samples from under-represented regions.

The VCS can be fully edited by the user to accommodate additional vaccine matching data generated locally for vaccine strains not already included in the tool. It is important to realize that antigenic match is not the only consideration regarding vaccination performance. Therefore, additional measures of vaccine performance could be considered, such as data from *in vivo* experiments or field vaccine evaluation studies, which are influenced by other important variables such as vaccine potency, vaccination regime and/or the weight of the infectious challenge (36, 37). The Supplementary Data displays results of published experimental *in vivo* studies that could be used to modify the VCS. Additionally, where vaccine matching data are not available, it is possible that alternative methods of measuring antigenic differences relevant to protection could be utilized, such as antigenic cartography (38) or sequence-based approaches (39).

PRAGMATIST relies on inputs provided by the user, the WOAH/FAO FMD Reference Laboratory Network and the WRLFMD for the SAS, LDS and VCS, respectively. The impact of uncertainty in these estimations (for SAS and LDS) or test variability (VCS) was assessed using sensitivity analyses. The introduction of uncertainty in the VCS resulted in a higher likelihood of change to the final vaccine scores, and therefore the final ranking of vaccine priority, in contrast to SAS and LDS which were more tolerant to a range of plausible input values without affecting the prioritization of the vaccine antigens. These findings demonstrated the importance of accommodating variability in vaccine matching and uncertainty where gaps in data exist into PRAGMATIST and motivate further effort to increase vaccine matching testing or access to data where possible, to improve confidence in these results, and to define the true profiles (distribution shape) for the VCS.

Ultimately, the vaccine score combines the LRS with the VCS, such that the highest scoring vaccines are those with the best antigenic match to the most prevalent lineages circulating in the highest risk source regions. However, when using the tool to assist with vaccine selection, the vaccine bank manager should also consider the diversity (breadth) of protection afforded by different vaccines and the need to choose a portfolio of complementary rather than overly redundant vaccine strains. Thus, if a vaccine provides a reliable match against a particular lineage (VCS close to 1 with many vaccine matching tests performed), adding additional vaccines to the vaccine bank will not provide additional protection against the risk from that specific lineage. For example, storage of O-3039 (BI) in addition to O/TUR/5/09 (MSD) would not provide additional protection against the risk from O/ME-SA/PanAsia-2, as these vaccines both have a high VCS, and a high number of vaccine matching tests were performed for these combinations. Therefore, it is not recommended to simply select the highest-scoring vaccines as these may provide redundant protection. However, the need for multiple vaccines is more obvious for serotype A due to the greater antigenic diversity within this serotype (33, 40). It should be noted that a low score for some vaccines may reflect a lack of vaccine matching testing rather than a lack of protection. For example, the O Panasia 2 (BI) vaccine only had vaccine matching results available for 3 of the 7 serotype O lineages (and only testing a maximum of 3 isolates), and thus any possible protection that may exist against the 4 untested lineages was not included in its final score. Other considerations that are not considered in PRAGMATIST but are likely to be important for vaccine antigen choice include the potency at which the vaccine can/will be provided, contractual arrangements with specific vaccine manufacturers, existing stock and expiration dates, and financial considerations. Finally, as described, the PRAGMATIST output is intended to inform vaccine selection given the current viral and incursion risks. However, the user could also parameterise the tool considering anticipated future risks, perhaps eventually applying bioinformatics to predict novel antigenic phenotypes of emerging strains and the protection conferred by current vaccine antigens.

In conclusion, vaccine bank holdings may be crucial to enable a swift and effective response to an incursion of FMD into a free country. Considering the complexity of different FMD vaccine antigens that are produced by different suppliers, PRAGMATIST was developed to support vaccine bank managers in this critical decision-making process, which is likely to have different outcomes depending on the geographical location. Due to the ever-changing dynamics of FMD virus circulation in endemic areas the tool should be updated on a regular basis to reflect the current situation and best data available. The focus of this paper was antigen bank management, and therefore the worked examples included vaccines from vaccine manufacturers that offer well-established antigen bank services. However, by making PRAGMATIST freely accessible in a dedicated, code-based, and highly customisable web-based dashboard, the tool is able to evolve and adapt to user needs, providing, for example, an option to add circulating strains as they are detected, or vaccines as they are developed, or to accommodate specific user's interests. Further, it is foreseen that a similar framework could incorporate heterologous serological data collected testing antisera to specific vaccine batches against regional virus threats. This would take account of both antigenic match and batch-specific vaccine potency in selecting FMD vaccines for preventative and emergency vaccination strategies in FMD endemic countries. Further efforts are also needed to increase the pool of useful matching data by closing surveillance gaps, sharing of material and inter-laboratory standardization of testing.

## Data availability statement

The original contributions presented in the study are included in the article/Supplementary material, further inquiries can be directed to the corresponding authors.

## Author contributions

MM, DK, AL, and MH designed the tool and/or the study. AL, MM, KP, and MH compiled the raw data. BA, JP, DH, and JC performed the data analysis for the manuscript and prepared the figures. AD, UM, and PM co-designed and developed the R Shiny interactive dashboard. BA, AL, MM, DK, AD, DP, KS, FR, JC, and JP were involved in preparation of the manuscript. All authors read, contributed, and approved the final manuscript.
