# Erratum: PRAGMATIST: A tool to prioritize foot-and-mouth disease virus antigens held in vaccine banks

**DOI:** 10.3389/fvets.2023.1143765

**Published:** 2023-01-26

**Authors:** Frontiers Production Office

**Affiliations:** Frontiers Media SA, Lausanne, Switzerland

**Keywords:** vaccination, vaccine matching, vaccine bank, foot and mouth disease (FMD), decision support tool, vaccine selection

Due to a production error, the copyright statement was published incorrectly. It was published as: “Copyright © 2022 Ludi, McLaws, Armson, Clark, Di Nardo, Parekh, Henstock, Muellner, Muellner, Rosso, Prada, Horton, Paton, Sumption and King. © FAO 2022. This is an open access article distributed under the terms of the Creative Commons Attribution IGO License, which permits unrestricted use, adaptation (including derivative works), distribution, and reproduction in any medium, provided the original work is properly cited. In any reproduction or adaptation of this article there should not be any suggestion that FAO or this article endorse any specific organization or products. The use of the FAO logo is not permitted. This notice should be preserved along with the article's original URL.”

The correct copyright statement should read as follows: “Copyright © Food and Agriculture Organization of the United Nations 2022. This is an open access article distributed under the terms of the Creative Commons Attribution IGO License (https://creativecommons.org/licenses/by/3.0/igo/legalcode), which permits unrestricted use, adaptation (including derivative works), distribution, and reproduction in any medium, provided the original work is properly cited. In any reproduction or adaptation of this article, there should not be any suggestion that the Food and Agriculture Organization of the United Nations, or this article, endorse any specific organization or products. The use of the Food and Agriculture Organization of the United Nations logo is not permitted. This notice should be preserved along with the article's original URL.”

The publisher apologizes for the mistake. The original article has been updated.

