# Supplementary material for: PRAGMATIST: A tool to prioritize foot-and-mouth disease virus antigens held in vaccine banks
Source: Front Vet Sci. 2022 Dec 15;9:1029075. doi: 10.3389/fvets.2022.1029075 (PMC9798001; doi:10.3389/fvets.2022.1029075)
Supplement: Supplementary file 1 [file Data_Sheet_1.zip › Data Sheet 2 (1).docx]

**Supplementary Data**

**Supplementary Table 1.** Results of *in-vivo* vaccine efficacy studies in the literature, which could be used to inform the vaccine coverage score.

| **Challenge virus** | **Vaccine (manufacturer)** | **Dose** | **Percentage of animals protected (number/total)*** | **Reciprocal log_10_ titre by VNT** | **Reference** |
| --- | --- | --- | --- | --- | --- |
| O/ME-SA/Ind-2001 | O Manisa (BI) | Various | 46.7 (7/15) | 3.0 (range 2.55–3.15) (protected animals) | (1) |
|  |  | Full (6PD_50_/dose) | 60.0 (3/5) |  |  |
|  |  | 1/4 | 80.0 (4/5) |  |  |
|  |  | 1/16 | 0.0 (0/5) |  |  |
| O/ME-SA/Ind-2001d (challenge at day 7) | O-3039 (BI) | Full (6PD_50_/dose) | 60.0 (3/5) | >1.0 | (2) |
| O/ME-SA/Ind-2001d (challenge at day 21) | O-3039 (BI) | Full (6PD_50_/dose) | 100.0 (5/5) | >2.0 | (2) |
| O/ME-SA/Ind-2001d (challenge at day 7) | O-3039 & O Manisa | Full (6PD_50_/dose) | 80.0 (4/5) | >1.0 | (2) |
| O/ME-SA/Ind-2001d (challenge at day 21) | O-3039 & O Manisa | Full (6PD_50_/dose) | 100.0 (5/5) | >2.0 | (2) |
| A/Asia/G-VII | A-IRN-05/A-SAU-95 (BI) | Full | 56.3 (9/16) | - | (3) |
| A/Asia/G-VII | A22/IRQ/64 (BI) | Full | 28.6 (2/7) | <1.5 | (4) |
| A/Asia/G-VII | A22Iraq24/64 (BI) |  |  |  | (5) |
| A/Asia/G-VII | A/MAY/97 (BI) | Full | 71.4 (5/7) | >1.5 | (4) |
|  | A/MAY/97 (BI) | Various | 86.7 (13/15) | <1.5 | (4) |
|  | A/MAY/97 (BI) | Full | 100.0 (5/5) | - | (4) |
|  | A/MAY/97 (BI) | 1/3 | 80.0 (4/5) | - | (4) |
|  | A/MAY/97 (BI) | 1/9 | 40.0 (2/5) | - | (4) |
| A/Asia/G-IX/SEA-97 | A22/IRQ/64 (BI) | Full (>6PD_50_/dose) | 100.0 (5/5) | 2.2 | (6) |
| A/Asia/G-IX/SEA-97 | A/MAY/97 (BI) | Full (>6PD_50_/dose) | 100.0 (5/5) | 2.0 | (6) |
| ASIA-1/ASIA/Sindh-08 | Asia 1 Shamir (BI) | Various | 86.7 (13/15) | - | Li *et al*.  (unpublished) |

*Cut-off for effective protection is defined as: 3/5 of animals protected with a log_10_ reciprocal titre of 1.5 after a single dose vaccination with serum collected 21 days later (according to the ‘Recommendation to AgResults on using serological indicators (“valency testing”) of cross-protection for FMD vaccines’)(7).

VNT: virus neutralisation test, BI: Boehringer Ingelheim.

**Supplementary Table 2.** Number of unique samples used for vaccine matching tests, per year of sample collection.

| Serotype/lineage | 2010 | 2011 | 2012 | 2013 | 2014 | 2015 | 2016 | 2017 | 2018 | 2019 | 2020 | 2021 | Total |
| --- | --- | --- | --- | --- | --- | --- | --- | --- | --- | --- | --- | --- | --- |
| O/CATHAY | 0 | 4 | 1 | 2 | 3 | 4 | 3 | 6 | 6 | 2 | 0 | 0 | 31 |
| O/EA or O/WA | 0 | 13 | 9 | 3 | 6 | 2 | 11 | 19 | 20 | 8 | 0 | 2 | 93 |
| O/EURO-SA | 0 | 1 | 0 | 0 | 0 | 0 | 0 | 0 | 0 | 0 | 0 | 0 | 1 |
| O/ME-SA/Ind-2001 | 0 | 0 | 4 | 12 | 10 | 8 | 11 | 17 | 12 | 9 | 5 | 2 | 90 |
| O/ME-SA/PanAsia | 0 | 5 | 11 | 5 | 3 | 6 | 2 | 2 | 7 | 1 | 0 | 0 | 42 |
| O/ME-SA/PanAsia-2 | 0 | 29 | 12 | 12 | 6 | 5 | 9 | 8 | 8 | 7 | 1 | 0 | 97 |
| O/SEA/Mya-98 | 0 | 5 | 5 | 4 | 12 | 7 | 9 | 3 | 3 | 3 | 0 | 0 | 51 |
| A/AFRICA | 0 | 4 | 8 | 11 | 0 | 2 | 2 | 7 | 11 | 3 | 0 | 1 | 49 |
| A/ASIA/G-VII | 0 | 0 | 0 | 0 | 0 | 5 | 4 | 9 | 1 | 0 | 0 | 0 | 19 |
| A/ASIA/Iran-05 | 0 | 16 | 12 | 18 | 9 | 9 | 4 | 7 | 3 | 2 | 2 | 0 | 82 |
| A/ASIA/Sea-97 | 0 | 4 | 8 | 11 | 4 | 12 | 11 | 6 | 1 | 4 | 0 | 0 | 61 |
| A/EURO-SA | 0 | 0 | 0 | 0 | 0 | 0 | 0 | 0 | 0 | 0 | 0 | 0 | 0 |
| Asia 1 | 0 | 18 | 12 | 5 | 7 | 6 | 3 | 6 | 2 | 4 | 0 | 0 | 63 |
| SAT 1 | 0 | 2 | 5 | 5 | 3 | 9 | 1 | 1 | 0 | 0 | 0 | 0 | 26 |
| SAT 2 | 1 | 6 | 18 | 2 | 5 | 10 | 0 | 4 | 4 | 4 | 2 | 0 | 56 |
| SAT 3 | 0 | 0 | 0 | 0 | 0 | 1 | 0 | 1 | 0 | 0 | 0 | 0 | 2 |
| Total | 1 | 107 | 105 | 90 | 68 | 86 | 70 | 96 | 78 | 47 | 10 | 5 | 763 |

**Supplementary Figures 1a-d.** Number of samples received per country, used for vaccine matching.

**Supplementary Figure 1a.** Typed as **s**erotype O. Not shown on the map: Nine samples typed as serotype O were also received for the Palestian Autonomous Territories.

**Supplementary Figure 1b.** Typed as serotype A. Not shown on the map: Three samples typed as serotype A were also received for the Palestian Autonomous Territories.

**Supplementary Figure 1c.** Typed as serotype Asia 1 or SAT 1.

**Supplementary Figure 1d.** Typed as serotype SAT 2. Not shown on the map: One sample typed as serotype SAT 2 was also received for the Palestian Autonomous Territories. Two samples from Zambia were also typed as SAT 3.

**Supplementary Figures 2a-b.** Sensitivity analysis using Source Area Scores for the North American and Australian vaccine bank perspectives.

Here, the additional aspects of the sensitivity analysis are shown, investigating the extent to which uncertainty in the input variables of the PRAGMATIST tool impacts the estimate of the risk covered. In the main text this example with the Source Area Scores (SAS) derived from expert elicitation from the European perspective is shown. There are also SAS from North America (Figure S2a) and Australia (Figure S2b), which are shown below. Only small differences in the overall risk ranking are observed, which was expected; and there is little variation associated with the SAS.

Supplementary Figure 2a. The risk covered by each vaccine is shown using mid-level confidence and North American Source Area Scores.

Supplementary Figure 2b. The risk covered by each vaccine is shown using mid-level confidence and Australian Source Area Scores.

**Supplementary Figures 3a-c.** Sensitivity analysis introducing the greatest amount of uncertainty in the SAS and LDS for each of the vaccine bank perspectives.

In the main text, the assertion is made that the vaccine coverage score has the greatest impact on the risk coverage. It is of note, however, that uncertainty was introduced differently for the vaccine scores, because there are two different sources of uncertainty that must be accounted for. As such, it is possible when using the mid-level uncertainty, that there is not a comparable amount of variation introduced to the SAS and Lineage Distribution Scores (LDS). Therefore, this is investigated, by setting confidence to none – introducing the greatest amount of uncertainty in the SAS and LDS. These are shown below for Europe (Figure S3a), North America (Figure S3b) and Australia (Figure S3c).

Supplementary Figure 3a. The percentage risk covered using European SAS, and the lowest levels of confidence (none) in the SAS and LDS scores.

Supplementary Figure 3b. The percentage risk covered using North American SAS, and the lowest levels of confidence (none) in the SAS and LDS scores.

Supplementary Figure 3c. The percentage risk covered using Australian SAS, and the lowest levels of confidence (none) in the SAS and LDS scores.

**References**

1. Fishbourne E, Ludi AB, Wilsden G, Hamblin P, Statham B, Bin-Tarif A, Brocchi E, Grazioli S, Dekker A, Eblé P, et al. Efficacy of a high potency O1 Manisa foot-and-mouth disease vaccine in cattle against heterologous challenge with a field virus from the O/ME-SA/Ind-2001 lineage collected in North Africa. *Vaccine* (2017) **35**:2761–2765. doi: 10.1016/j.vaccine.2017.02.047

2. Singanallur NB, Dekker A, Eblé PL, van Hemert-Kluitenberg F, Weerdmeester K, Horsington JJ, Vosloo W. Emergency FMD Serotype O Vaccines Protect Cattle against Heterologous Challenge with a Variant Foot-and-Mouth Disease Virus from the O/ME-SA/Ind2001 Lineage. *Vaccines* (2021) **9**:1110. doi: https:// doi.org/10.3390/vaccines9101110

3. Waters R, Ludi AB, Fowler VL, Wilsden G, Browning C, Gubbins S, Statham B, Bin-Tarif A, Mioulet V, King DJ, et al. Efficacy of a high-potency multivalent foot-and-mouth disease virus vaccine in cattle against heterologous challenge with a field virus from the emerging A/ASIA/G-VII lineage. *Vaccine* (2018) **36**:1901–1907. doi: 10.1016/j.vaccine.2018.02.016

4. Dekker A, Sanz-Bernardo B, Singanallur NB, Ludi AB, Horsington J, Eblé PL, King DP, Vosloo W. Cross-protection induced by a A/MAY/97 emergency vaccine against intra-serotype heterologous challenge with a foot-and-mouth disease virus from the A/ASIA/G-VII lineage. *Vaccines* (2020) **8**:1–17. doi: 10.3390/vaccines8010024

5. Brehm KE, Kumar N, Thulke HH, Haas B. High potency vaccines induce protection against heterologous challenge with foot-and-mouth disease virus. *Vaccine* (2008) **26**:1681–1687. doi: 10.1016/j.vaccine.2008.01.038

6. Nagendrakumar B, Dekker A, Eblé PL, van Hemert-Kluitenberg F, Weerdmeester K, Horsington J, Wilna VW. Emergency foot-and-mouth disease vaccines a Malaysia 97 and A22 Iraq 64 offer good protection against heterologous challenge with a variant serotype a ASIA/G-IX/SEA-97 lineage virus. *Vaccines* (2020) **8**:1–16. doi: 10.3390/vaccines8010080

7. WRLFMD. Recommendation to AgResults on using serological indicators (“valency testing”) of cross protection for FMD vaccines. https://www.wrlfmd.org/sites/world/files/quick_media/Cross-neutralisation measure AgResults Final v2.1.pdf
