# Supplementary figures and images for: PRAGMATIST: A tool to prioritize foot-and-mouth disease virus antigens held in vaccine banks
Source: Front Vet Sci. 2022 Dec 15;9:1029075. doi: 10.3389/fvets.2022.1029075 (PMC9798001; doi:10.3389/fvets.2022.1029075)

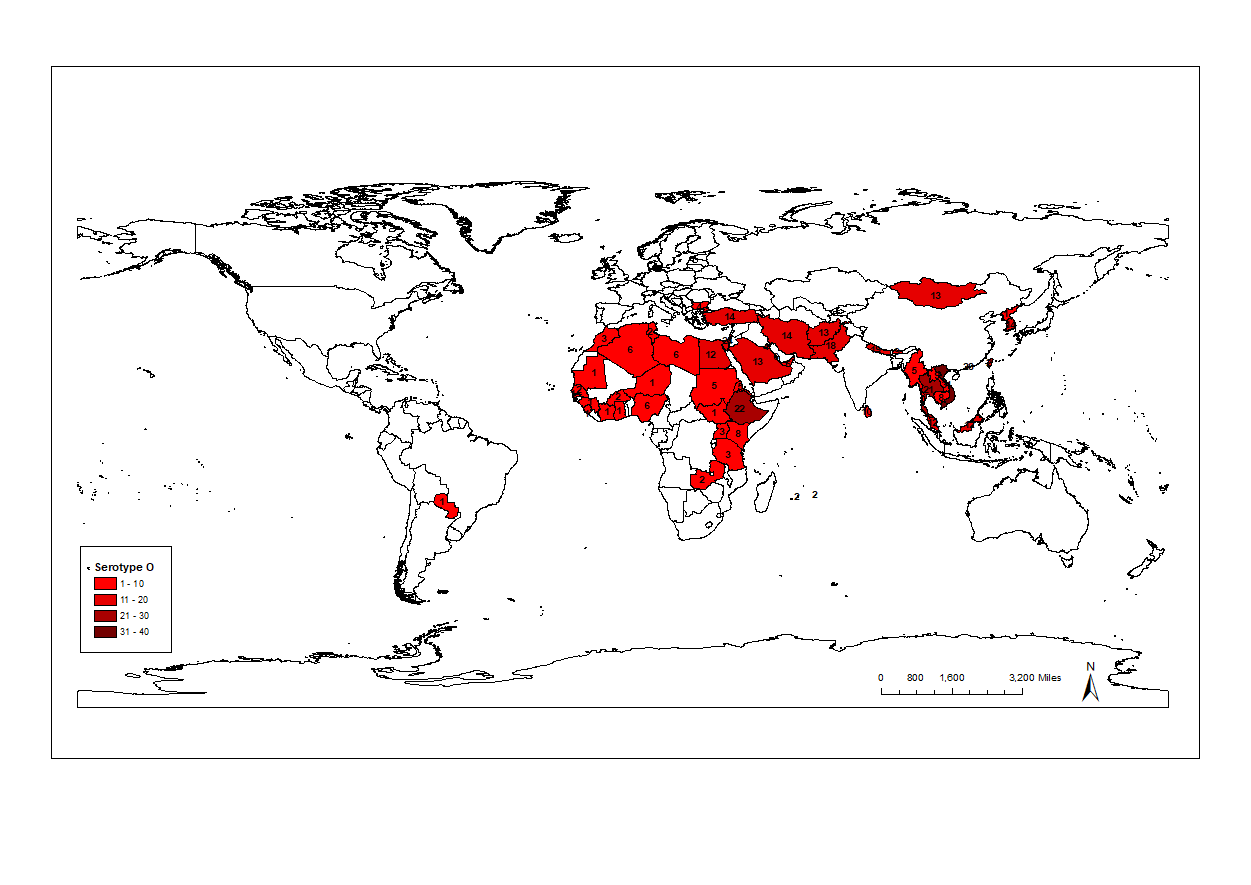

Supplement: Supplementary file 1 [file Data_Sheet_1.zip › Supplementary Figure 1a.TIF]

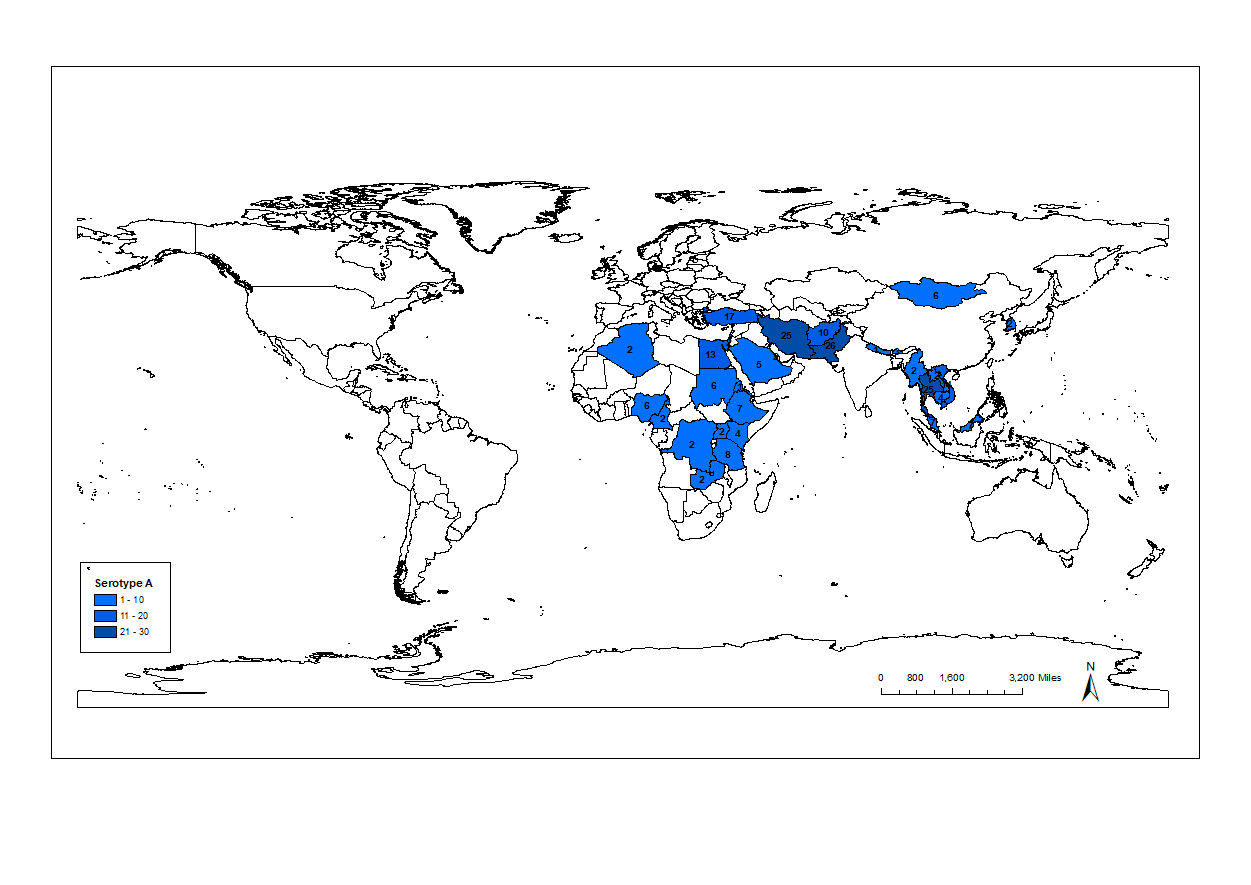

Supplement: Supplementary file 1 [file Data_Sheet_1.zip › Supplementary Figure 1b.TIF]

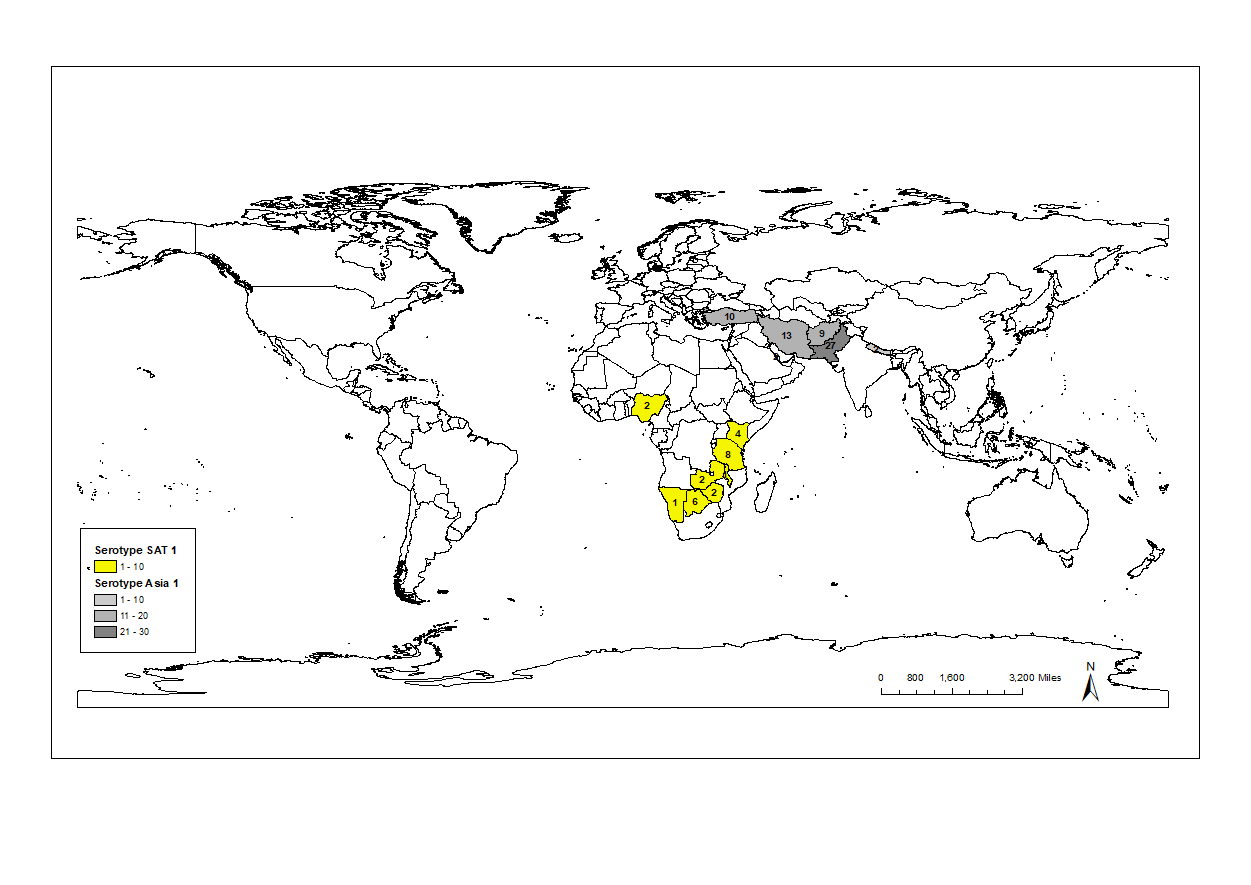

Supplement: Supplementary file 1 [file Data_Sheet_1.zip › Supplementary Figure 1c.TIF]

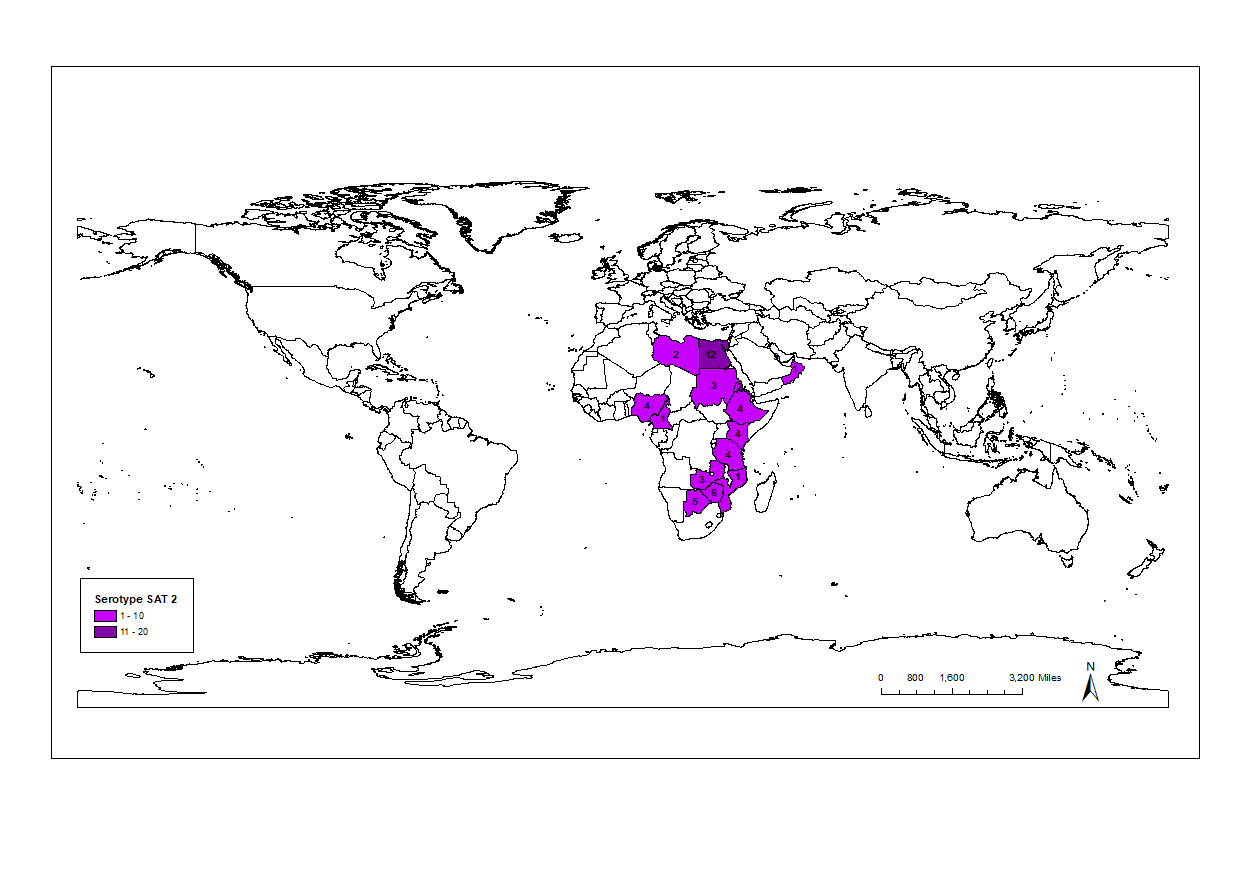

Supplement: Supplementary file 1 [file Data_Sheet_1.zip › Supplementary Figure 1d.TIF]

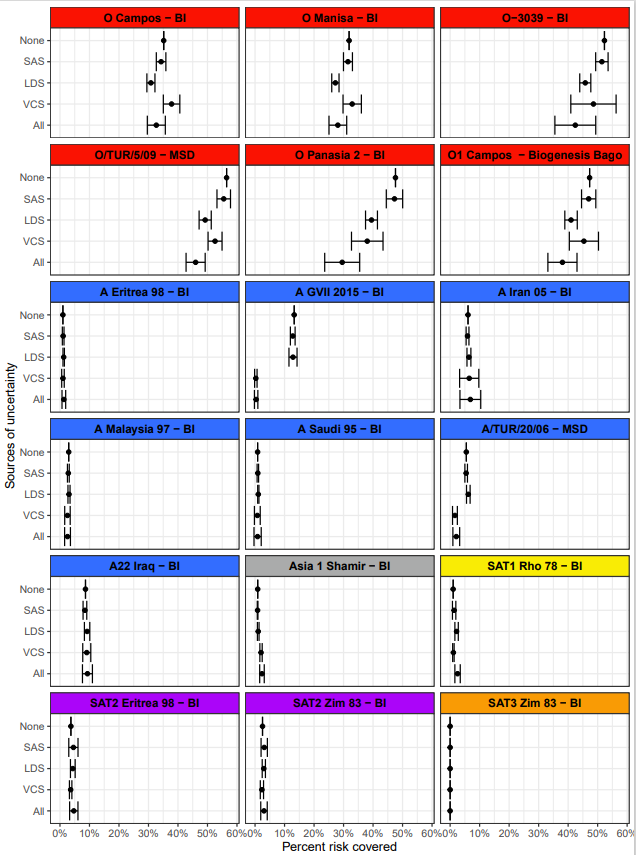

Supplement: Supplementary file 1 [file Data_Sheet_1.zip › Supplementary Figure 2a.TIF]

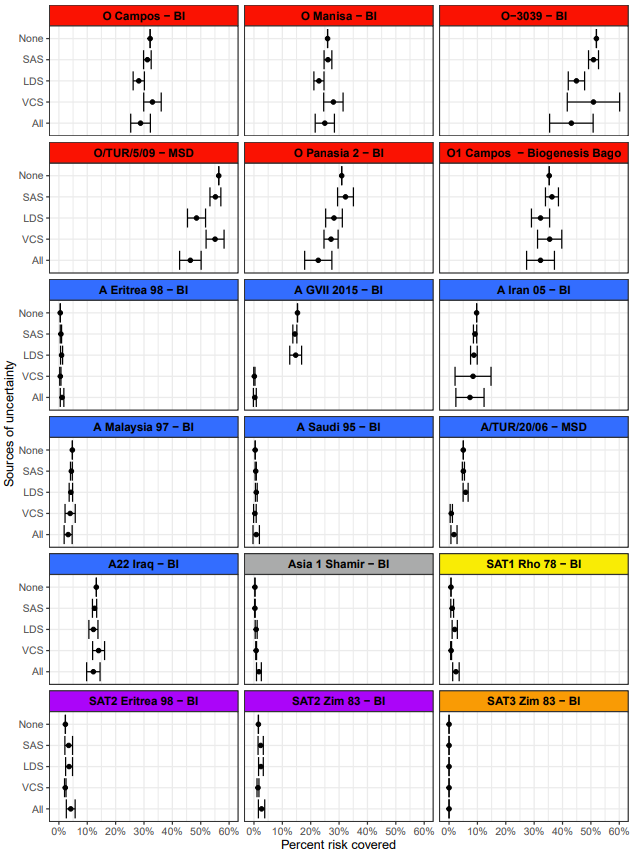

Supplement: Supplementary file 1 [file Data_Sheet_1.zip › Supplementary Figure 2b.TIF]

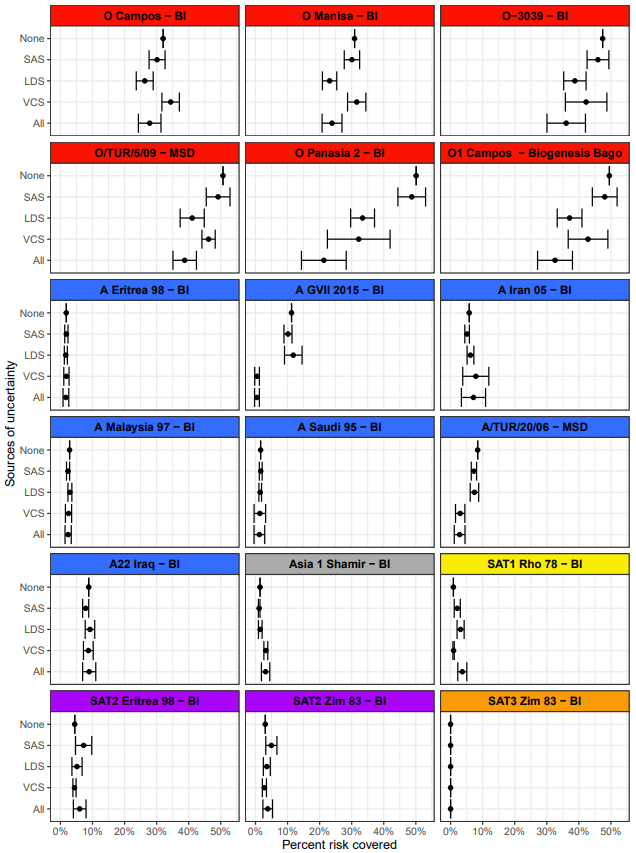

Supplement: Supplementary file 1 [file Data_Sheet_1.zip › Supplementary Figure 3a.TIF]

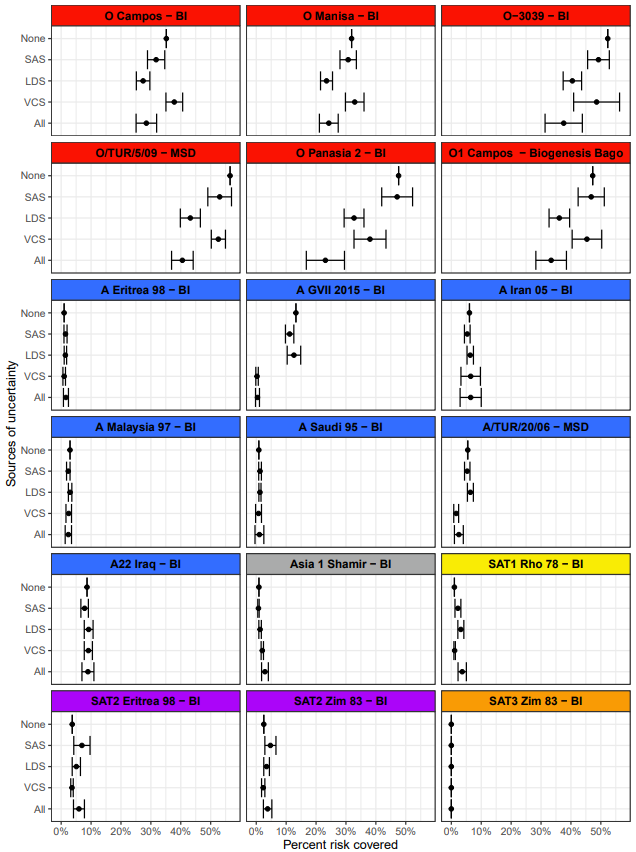

Supplement: Supplementary file 1 [file Data_Sheet_1.zip › Supplementary Figure 3b.TIF]

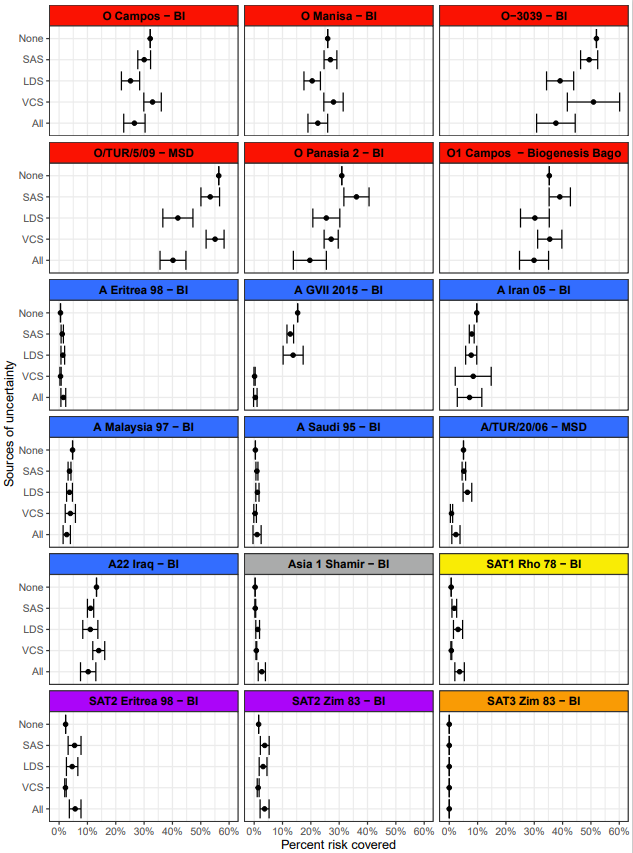

Supplement: Supplementary file 1 [file Data_Sheet_1.zip › Supplementary Figure 3c.TIF]
